# Exploring the Relationship between Melioidosis Morbidity Rate and Local Environmental Indicators Using Remotely Sensed Data

**DOI:** 10.3390/ijerph21050614

**Published:** 2024-05-13

**Authors:** Jaruwan Wongbutdee, Jutharat Jittimanee, Suwaporn Daendee, Pongthep Thongsang, Wacharapong Saengnill

**Affiliations:** 1 Geospatial Health Research Group, College of Medicine and Public Health, Ubon Ratchathani University, Ubonratchathani 34190, Thailand; jaruwan.w@ubu.ac.th (J.W.); jutharat.j@ubu.ac.th (J.J.); suwaporn.d@ubu.ac.th (S.D.); 2 Department of Geology, Faculty of Science, Chulalongkorn University, Bangkok 10330, Thailand; pongthep.t@chula.ac.th

**Keywords:** geographically weighted Poisson regression, Google Earth engine, spatial model, *Burkholderia pseudomallei*

## Abstract

Melioidosis is an endemic infectious disease caused by *Burkholderia pseudomallei* bacteria, which contaminates soil and water. To better understand the environmental changes that have contributed to melioidosis outbreaks, this study used spatiotemporal analyses to clarify the distribution pattern of melioidosis and the relationship between melioidosis morbidity rate and local environmental indicators (land surface temperature, normalised difference vegetation index, normalised difference water index) and rainfall. A retrospective study was conducted from January 2013 to December 2022, covering data from 219 sub-districts in Northeast Thailand, with each exhibiting a varying morbidity rate of melioidosis on a monthly basis. Spatial autocorrelation was determined using local Moran’s *I*, and the relationship between the melioidosis morbidity rate and the environmental indicators was evaluated using a geographically weighted Poisson regression. The results revealed clustered spatiotemporal patterns of melioidosis morbidity rate across sub-districts, with hotspots predominantly observed in the northern region. Furthermore, we observed a range of coefficients for the environmental indicators, varying from negative to positive, which provided insights into their relative contributions to melioidosis in each local area and month. These findings highlight the presence of spatial heterogeneity driven by environmental indicators and underscore the importance of public health offices implementing targeted monitoring and surveillance strategies for melioidosis in different locations.

## 1. Introduction

The distribution of melioidosis, a bacterial infection caused by *Burkholderia pseudomallei*, can be examined in relation to the soil [1,2,3] and water resources [4]. Rainfall, soil water surfaces, and flooding are commonly associated with an increased incidence of melioidosis [5,6]. Melioidosis infections can be caused by the inhalation, skin abrasion, and ingestion of *B. pseudomallei* [4,7]. *B. pseudomallei* is found in soil depths of 0–90 cm [8], having an optimal growth temperature of 37 °C [9]. It can survive in soil moisture of more than 10%, lasting for over a year at a 20% survival rate [10,11]. 

Changes in climate and environmental conditions lead to changes in health status, health-related illnesses, and death [12,13]. They also influence the transmission of infectious diseases such as melioidosis [14]. Epidemiology, the study of the distribution and determinants of health status or health-related events in a given population, puts the results of various studies to use for the protection and control of health problems. Monitoring and collecting environmental data over long periods can support the development and planning of disease prevention and surveillance programmes. These require the presentation of spatiotemporal disease occurrence. Remote sensing is used to detect and track the physical characteristics of an area, such as land surface temperature (LST), normalised difference vegetation index (NDVI), and normalised difference water index (NDWI), by measuring reflected and emitted radiation from a distance. To analyse the relationship of these indices with diseases, such as dengue, malaria, and leptospirosis, high-resolution satellite images are assigned pixel values for each index [15,16,17]. 

Currently, studies on melioidosis disease have only been limited to field remote sensing. Geospatial data, which are data with georeferenced coordinates to positions on the Earth’s surface, have been utilised to monitor and investigate the risk area of melioidosis occurrence. Several studies have conducted spatial analyses of melioidosis distribution in the endemic regions of Australia [2,18], Thailand [19,20,21], and Laos [22]. Geostatistical modelling has revealed that the range distance of the spatial autocorrelation in a quantitative *B. pseudomallei* count was 7.6 m [23], and the range distance between positive *B*. *pseudomallei* samples was 90.51 m in a rice field [24]. To elucidate the conditions in which melioidosis infections can be increased in humans, an environmentally optimal bacterium, such as *B. pseudomallei,* positive can be used. 

The Google Earth engine (GEE) is a cloud-based platform that revolutionises environmental analyses by granting access to vast collections of satellite imagery and geospatial data, empowering researchers to study diverse environmental factors such as land cover changes, climate patterns, and ecosystem dynamics. Leveraging its capabilities in time-series analysis, data visualisation, and algorithm development, the GEE facilitates the monitoring of global-scale environmental changes over time. It can be effectively used to study infectious diseases by integrating geospatial data and advanced analytics. For vector-borne diseases such as malaria, dengue fever [25,26], and COVID-19 [27,28], the platform can play a crucial role in understanding disease dynamics and improving public health interventions. In the case of melioidosis, a study revealed the spatial distribution pattern of melioidosis incidence using local Moran’s *I* and its spatial risk area using indicator interpolation kriging [21]. However, this study only considered a single variable. Furthermore, the limited information in a local area is not sufficient for understanding the characteristics and potential of melioidosis infection in other locations. Therefore, different environmental indicators in a local area should be utilised to reveal their relationships with melioidosis outbreaks. 

To this end, this study aimed to investigate the relationship of melioidosis morbidity rate with local environmental indicators, specifically LST, NDVI, NDWI, and rainfall using remote sensing data and geographically weighted Poisson regression (GWPR). The objectives of this study were to (1) determine the spatiotemporal dependence of melioidosis distribution and identify the monthly hot and cold spots and (2) classify the monthly data of melioidosis morbidity rate and the environmental indicators over a period of 10 y. The results of this study will be beneficial for the spatial monitoring and surveillance of melioidosis outbreaks in local areas.

## 2. Data and Methods

### 2.1. Study Area

This study was conducted in the Ubon Ratchathani province in Northeast Thailand. The province has an area of 15,774 km^2^ and covers 25 districts, which are further divided into 219 sub-districts. It is adjacent to the borders of Cambodia and Laos and hosts three significant rivers: Mun, Chi, and Mekong. The study area experiences three seasons: summer, which begins in mid-February and lasts until mid-May; the rainy season, which extends from mid-May to mid-October; and winter, which is influenced by the northeastern monsoon winds, beginning in mid-October and lasting until mid-February. The morbidity rate of melioidosis in the area was obtained from the National Disease Surveillance (Report 506), revealing a morbidity rate of 29.18 per 100,000 people in 2019 and 25.71 per 100,000 people in 2020 [29,30].

### 2.2. Conceptual Framework

**Figure 1:**
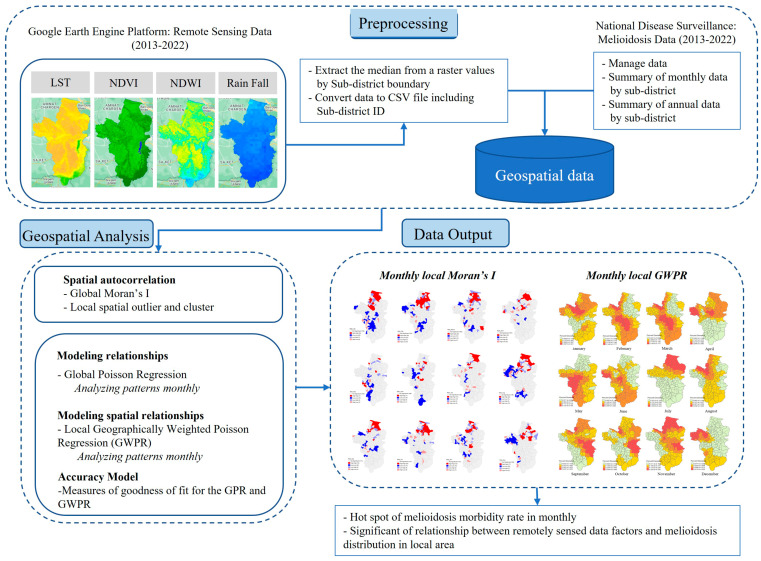
Conceptual framework of the study.

This was an applied study of the spatiotemporal dynamics of provincial melioidosis outbreaks. It shows a map of sub-district boundaries using long-term data over a 10 y period, showing monthly outbreak periods. Spatial autocorrelation was used to determine the spatial distribution pattern, which showed clusters, randomness, and dispersion. The local spatial outlier and cluster revealed the location of the melioidosis hotspots in the study area. We required the understanding of local spatial indicators to track and determine the ones that influence melioidosis. This study used big data from satellite images at multiple time periods and scales through the GEE platform. Data were accessed by writing JavaScript commands and filtering the date and then reducing the map using median values following the sub-district area. Spatial data were extracted from each sub-district and exported as a CSV file. The data were then linked to the spatial indicators (LST, NDVI, NDWI, and rainfall) and melioidosis according to the location of the sub-district. GWPR analysis was used to determine the relationship between spatial indicators and the melioidosis morbidity rate in the local area for each month, as shown in Figure 1. The software used for analysing and visualising the spatial model were as follows: (1) spatial autocorrelation (Moran’s *I*) using Geoda v.1.20.0.22, (2) GWPR using AcrGIS Pro 3.2.0, and (3) map visualisation using R v.4.2.1 with the tmap package [31].

### 2.3. Melioidosis Data

The 10 y attribute data from 1 January 2013 to 31 December 2022, which included melioidosis case reports, were obtained from the public health office in Ubon Ratchathani. A data frame, which is a two-dimensional data structure aligned in a tabular manner in rows and columns, was created. The columns included the sex, age, occupation, Tambon ID (sub-district ID), and date of melioidosis cases confirmed by a doctor at the hospital. Then, the data were mutated, and the rows that were missing or not related to the study area were excluded. The morbidity rate per 10,000 people was calculated by dividing the number of melioidosis cases by the sub-district and number of people in the sub-district. Subsequently, the attribute data were connected one-to-one to the map of the sub-district boundary using the field Tambon ID. Geographical coordinates were based on the Universal Transverse Mercator (UTM) system zone 48N (EPSG 32648). Human protocols were approved by The Ubon Ratchathani University-Human Ethic Committee (ID# UBU-REC-171/2565).

### 2.4. Spatial Data

The GEE is a cloud-based system for processors, data storage, satellite remote sensing analysis, and other environmental and climate data products. The big data of high-resolution satellite images are freely accessible online via the JavaScript, Python, and R computer languages. By analysing the data collected from remote sensing technologies, the environmental indicators associated with the occurrence of a disease can be investigated within a specific locality. 

In this study, the use of spatiotemporal analyses allows for a comprehensive understanding of the patterns and trends in the distribution of melioidosis over time. Moreover, the integration of spatial data enhanced our ability to explore the temporal dynamics of this disease and its relationship with various environmental indicators, namely LST, NDVI, NDWI, and rainfall. The application of remotely sensed data in this context provides valuable insights into the complex interactions between melioidosis and local environmental indicators that contribute to its spread. Overall, this approach enabled a detailed examination of the spatiotemporal aspects of melioidosis distribution, which can facilitate the identification of potential risk factors and the development of effective prevention and control strategies. The environmental factors are shown in Table 1.

#### 2.4.1. LST

The MOD11A1.006 product of the Moderate Resolution Imaging Spectroradiometer (MODIS) dataset provides daily global information on terrestrial LST and emissivity. Operated on the Terra satellite, this product has a high spatial resolution of 1 km. Emissivity information is particularly valuable for correcting temperature values based on the characteristics of different land surface types. Overall, the MOD11A1.006 product is a valuable resource for understanding the daily LST variations on a global scale.

#### 2.4.2. Vegetation

The NDVI was acquired from the MOD13Q1 V.6 product, providing 16 d surface reflectance data for vegetation in the red (ρRED) and near-infrared (ρNIR) channels at a resolution of 250 m. NDVI is calculated using the formula: NDVI = (ρNIR − ρRED)/(ρNIR + ρRED). The contrast between the RED and NIR responses serves as a sensitive indicator of vegetation abundance, with maximum differences observed over areas with a full canopy and minimal contrast over regions lacking vegetation. In areas with low to medium vegetation density, the contrast results from changes in both the RED and NIR channels, whereas in areas with high vegetation density, the increase in contrast is primarily attributed to changes in the NIR channel as the RED band becomes saturated owing to chlorophyll absorption [32].

#### 2.4.3. Soil Moisture

The NDWI is a satellite-derived metric from the ρNIR and Short-wave Infrared (ρSWIR) channels, designed for the estimation of water content within internal leaf structures. Employing the MOD09GA V.6 data, updated daily at a resolution of 463.313 m by MODIS and subjected to cloud cover masking, the NDWI is computed using the formula: NDWI = (ρNIR − ρSWIR)/(ρNIR + ρSWIR) [33,34]. The SWIR channel captures alterations associated with vegetation water content and spongy mesophyll structure, whereas the NIR channel responds to leaf internal structure and dry matter content, excluding considerations for water content [34]. Through the amalgamation of NIR with SWIR, the index efficiently eliminated variations induced by leaf structure and dry matter, yielding a more precise evaluation of vegetation water content. The resultant NDWI product, expressed as a dimensionless value within the range of −1 to +1, not only signifies leaf water content but also provides valuable insights into the vegetation type and cover.

#### 2.4.4. Rainfall

The Climate Hazards Group InfraRed Precipitation with Station (CHIRPS) data is a quasi-global (50 S–50 N), land-only rainfall dataset characterised by diverse spatiotemporal resolutions. Operating at a resolution of 5566 m, the data contain amalgamated information from various sources, including ground station measurements and satellite data. An extension of a previously established climatology dataset, CHIRPS, leverages satellite information to fill data gaps in areas that lack ground station data. The dataset was constructed by integrating daily and monthly infrared cold cloud duration (CCD) precipitation estimates using an innovative blending procedure to incorporate the spatial correlation structure of the CCD. Acknowledging its utility on a global scale [35], the CHIRPS has been instrumental in assessing and monitoring precipitation patterns. Notably, CHIRPS has been employed to evaluate rainfall in various regions of Thailand [36,37,38].

### 2.5. Data Preparation and Pre-Processing

**Figure 2:**
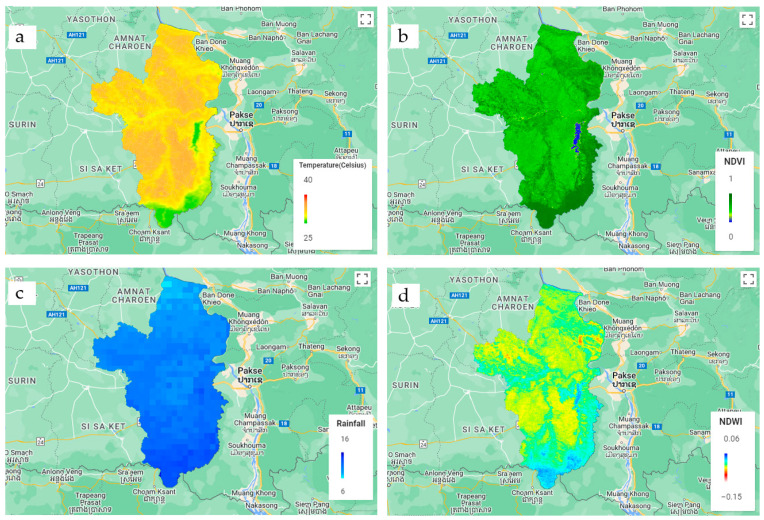
Median values of the remotely sensed data in 2013–2022 for (**a**) land surface temperature (LST), (**b**) normalised difference vegetation index (NDVI), (**c**) rainfall, and (**d**) normalised difference water index (NDWI).

Remotely sensed data were meticulously selected and filtered based on the geographical area and date, employing the capabilities of a JavaScript API. Satellite data collection involved clipping and extension procedures aligned with district boundaries (Figure 2). Monthly median descriptive statistics were applied to reduce the pixel values within each tambon boundary. For rainfall data obtained from the CHIRPS product, the images were resampled to a 1000 m resolution. Subsequently, data comprising the four environmental indicators were exported into a CSV file. This comprehensive dataset covered 219 administrative boundaries within Ubon Ratchathani. To establish spatial relationships, attribute data were connected using sub-district codes, linking melioidosis morbidity rate to environmental parameters. This method facilitated the creation of spatial data, enabling a nuanced exploration of the interplay between melioidosis and the chosen environmental indicators.

### 2.6. Spatial Statistics

#### 2.6.1. Spatial Autocorrelation

Spatial autocorrelation, which examines the similarity among observation values at different locations, reveals distribution patterns, such as clustering, dispersion, and random distribution. In our investigation of the melioidosis morbidity rate, we utilised the global Moran’s *I* statistic with queen contiguity-based weights, signifying a shared boundary edge between spatial units. Neighbouring relationships are assigned values of 1 or 0. Moran’s *I* ranges from −1 to 1, where positive values denote clustering, negative values indicate dispersion, and 0 suggests a random distribution. The statistical significance was set at a pseudo *p* < 0.05.

The utilisation of Local Moran’s *I* in our study allowed for the identification of hotspots, cold spots, and spatial outliers by evaluating neighbouring relationships, providing insights into zones with either high or low morbidity rates and their spatial connections. Cluster and outlier detection, employing four autocorrelation types, was complemented by the Local Indicators of Spatial Association technique, which further scrutinised spatial patterns, emphasising districts with similar morbidity rates surrounded by similar districts. Positive values assigned to features with high or low values among neighbours, along with the assessment of dissimilarity with neighbouring *I* values using negative values, contributed to a comprehensive understanding of the spatial dynamics. The incorporation of z-scores and *p*-values aided in evaluating the null hypothesis for significance and determining the output feature class for spatial dependency. This holistic approach provided a nuanced examination of the spatial distribution of the morbidity rate of melioidosis in various zones. 

#### 2.6.2. Global Poisson Regression (GPR)

A GPR model was used to examine the overall relationship between the morbidity rate of melioidosis and the environmental indicators in the study area. Before integrating the variables into the equation for GPR (Equation (1)), multicollinearity among the predictors was analysed to evaluate their independence. This analysis employed the variance inflation factor (VIF); values below 7.5 indicated non-multicollinearity and were consequently included in the model.

(1) $$ln(y_{i})=ln(\beta_{0})+\sum_{k=1}^{p}\beta_{k}X_{ki}\;$$

where ${ln(y}_{i})$ is the expected value of melioidosis morbidity rate in the tambon *I*, $\beta_{0}$ is the global intercept, $X_{ki}$ is the *k*th explanatory variable, and $\beta_{k}$ is the parameter estimating the explanatory variables.

#### 2.6.3. Local Poisson Regression

The GWPR is a spatial statistical technique used in the analysis of spatially distributed count data, where the outcome variable represents the number of events or occurrences in a given area. This method is an extension of the traditional Poisson regression, which considers the spatial heterogeneity of the data. The GWPR method was employed to address spatial relationships and instability issues, specifically concerning count and ordinary data. Utilising an analytical framework derived from the GWR model [39], the GWPR extends its application to investigate the correlation between disease occurrence and spatial variability. This model, a form of conditional kernel regression, utilises spatial weighting functions to estimate locally varying coefficients within the Poisson regression parameters. This approach allowed the creation of local surface maps illustrating spatial fluctuations in the relationship between the monthly melioidosis morbidity rate and localised distribution across the tambons. Consequently, this methodology facilitated the identification and in-depth exploration of locations that exhibited correlated relationships with the spatial indicators of LST, NDVI, NDWI, and rainfall. The GWPR model was formulated using Equation (2).

(2) $$ln(y_{i}\left(u_{i},\;v_{i}\right))=ln(\beta_{0}\left(u_{i},\;v_{i}\right))+\sum_{k=1}^{p}\beta_{k}\left(u_{i},\;v_{i}\right)X_{ki}\;$$

where *Y* represents the expected value of the melioidosis morbidity rate at the coordinate location, $\left(u_{i},\;v_{i}\right)$ denotes the two-dimensional coordinates of the *i*th tambon, and $\beta_{0}$ and $\beta_{k}$ represent the locally estimated intercept and the effect of variable *k* for location *i,* respectively. 

The coefficients $\hat{\beta }\left(u_{i},\;v_{i}\right)$ were calculated using Equation (3) through spatial weighting. They were weighted by distance based on observations in nearby tambons, with data from tambons closer to the point being weighted by fixed and adaptive kernels. The optimal distance influenced the observed location, which was determined by the size of the nearest spatial unit.

(3) $$\hat{\beta }\left(u_{i},\;v_{i}\right)={\left(x^{T}w\left(u_{i},\;v_{i}\right)x\right)}^{-1}x^{T}\;w\left(u_{i},\;v_{i}\right)Y$$

where w is an n-by-n geographical weight matrix of the sub-districts.

(4) $$w_{ij}=f\left(x\right)=\left\{\begin{array}{ll} {\left[1-{\left(\frac{\left\|u_{i}-v_{j}\right\|}{G_{i}}\right)}^{2}\right]}^{2} & ,\;\;\;\;\;\left\|u_{i}-v_{j}\right\|<G_{i}\\ 0 & ,\;\;\;\;\;otherwise \end{array}\right.$$

where $w_{ij}$ is the spatial geographical weight, $\left\|u_{i}-v_{j}\right\|$ represents the Euclidean distance between the tambon *i* and *j*, and $G_{i}$ represents the adaptive bandwidth size.

In the fixed kernel type, the data were weighted by a measure of the distance from the calibration location, considering data limitations. In the adaptive kernel type, the number of neighbouring tambons was optimised for each geographical region. Generally, Gaussian and bi-square kernel functions are used to produce a weight scheme when defining spatial units. We opted for a bi-square function with an adaptive kernel to estimate the spatial weights in the GWPR model based on the suggestions from previous research [39,40,41]. The optimal bandwidth was determined by lower AICc values, indicating the best model fit. Therefore, the AICc was also used to evaluate the model performance and compare the goodness-of-fit measurements between GPR and GWPR. Additionally, we considered the percentage of deviance explained as a measure of how well the model fit, indicating whether the explanatory variables can explain the relationship with the melioidosis morbidity rate. Subsequently, the residuals of the GWPR regression model were assessed for spatial correlations among adjacent tambons using Moran’s *I*. If the spatial autocorrelation value was close to 0, the null hypothesis was accepted, indicating a perfectly random spatial pattern with a significance level of 0.05.

## 3. Results

### 3.1. Melioidosis Morbidity Rate

**Figure 3:**
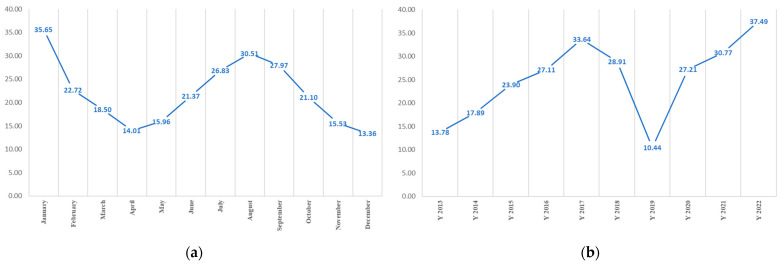
Melioidosis morbidity rate by (**a**) month and (**b**) year.

Between 2013 and 2022, a comprehensive analysis of melioidosis cases, which totalled 4871, was conducted. Gender classification revealed 3262 cases among males and 1609 among females. Predominantly, those affected were farmers (1943), followed by hired individuals (258). The monthly morbidity rates exhibited distinctive patterns, with January recording the highest rate, followed by August and September (Figure 3a). A closer examination of the monthly trends in the morbidity rate of melioidosis from January to December revealed that January had the highest morbidity rate, which gradually decreased until April. Conversely, morbidity rates increased from June to August, followed by a gradual decline until December. These findings provide valuable insights into the seasonal dynamics of melioidosis over a specified period.

As shown in Figure 3b, among the annual melioidosis rates between 2013 and 2022, 2022 recorded the highest rate, followed by 2017 and 2021, whereas 2019 marked the lowest rate. Notably, melioidosis rates exhibited a rapid increase from 2013 to 2017, averaging 4.96. Subsequently, a substantial decline occurred from 2017 to 2019, which represented the lowest rate in the decade. However, the morbidity rate experienced a notable resurgence, reaching 37.49 in 2022.

### 3.2. Spatial Autocorrelation

Table 2 illustrates the global Moran’s *I* values depicting the spatial autocorrelation pattern of the monthly melioidosis morbidity rate from 2013 to 2022, highlighting the spatial distribution clustering in the study area. January exhibited the highest spatial correlation. In contrast, the correlation gradually decreased from February to June, followed by a general upward trend, reaching 0.257 in December. The Local Moran’s *I* results pinpointed the spatial clusters, indicating the hot and cold spots of melioidosis morbidity rate. 

**Figure 4:**
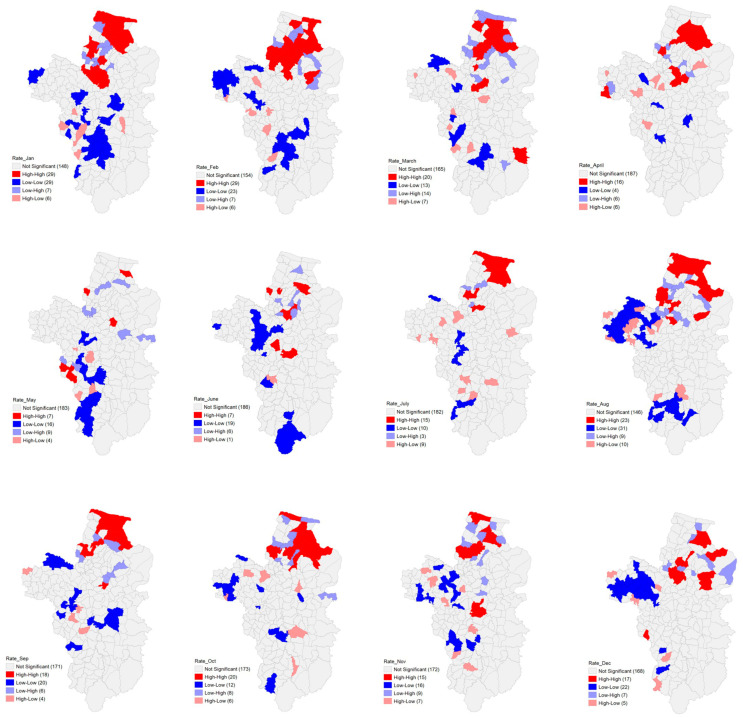
Spatial autocorrelation of melioidosis morbidity rate per month.

Spatial hotspots emerged predominantly in the northern part of the study area. The most prevalent spatial cluster (high–high) occurred in January and February, encompassing 29 tambons, followed by August with 23 tambons, and March and October with 20 tambons each. Notably, hotspots in May and June were scattered across the central and northern regions (Figure 4).

### 3.3. GPR Model

The GPR model associated with cover area represented the intercepts and coefficients of explanatory variables at a statistical significance of *p* ≤ 0.05 (Table S1). All explanatory variables were selected for the regression analysis because all values ignored multicollinearity when the VIF was <5. The results of the parameters in the GPR model are listed in Table S1. The statistically significant (*p* ≤ 0.05) months related to the morbidity rate of melioidosis were August, July, and January. In August, all explanatory variables affected the melioidosis morbidity rate; the intercept and NDVI were negatively significant, whereas LST, NDWI, and rainfall were positively significant. Additionally, LST was not significant in January, whereas the other variables were significant at *p* ≤ 0.05. In July, rainfall was excluded from the model.

### 3.4. Local Poisson Regression

The summary of the descriptive statistics (minimum, median, and maximum) of the coefficients were based on the GWPR model results (Table S1). Table 3 shows the median coefficients representing the estimated impact of environmental indicators on the response variable, which is likely related to the occurrence of melioidosis. A negative intercept coefficient suggests a baseline reduction in the expected count. A positive LST coefficient indicates that higher temperatures were associated with the increase in the expected count of the response variable. A positive NDVI coefficient implies that areas with higher vegetation density corresponded to higher expected counts. A negative NDWI coefficient suggests a negative association between the water index and response variable. A positive rainfall coefficient indicates that increased precipitation was linked to higher expected counts. The values of these coefficients provide insight into the spatially varying relationships between environmental indicators and health-related outcomes, offering a nuanced understanding of the geographical complexities involved in the study. In addition, GWPR calibrated the association between the melioidosis morbidity rate and LST, NDVI, NDWI, and rainfall for individual tambons.

### 3.5. Local Percent Deviance

**Figure 5:**
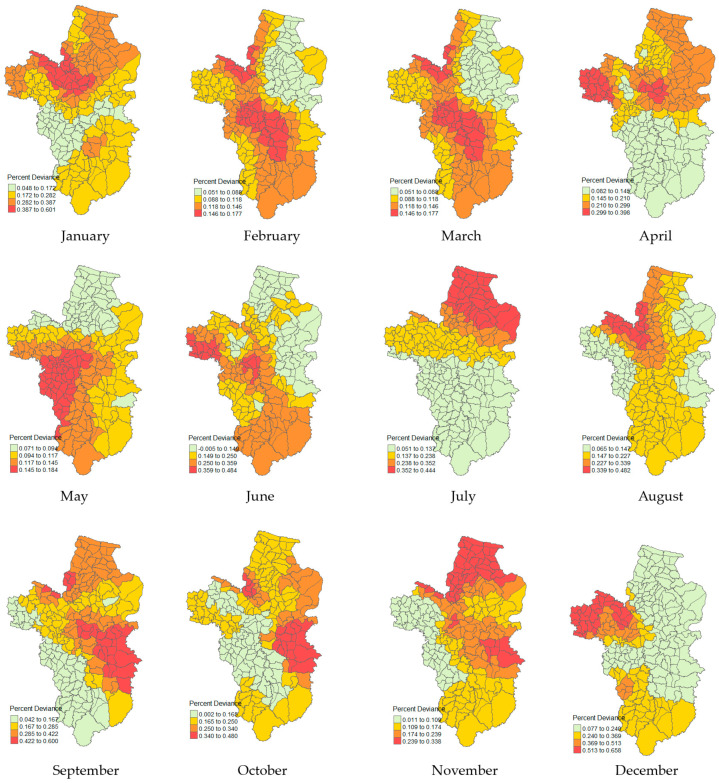
Local percent of deviance explained by the GWPR model.

Figure 5 shows the local percentage of the deviance-explained map that represents a potential relationship and fit accuracy of the explanatory variables that drive the changes in the melioidosis morbidity rate. The spatial distribution of the percent deviance varies over time and space every month, which was inherently grouped by the Jenks natural breaks classification. The red colour on the map indicates a higher value of local percentage deviance, showing a clear pattern of spatial variation. The maximum local accuracy ranges were 0.600–0.658 in January, September, and December, respectively, with the highest obtained in December. In December, the risk areas were mostly located in the western region (29 tambons). In January, a trend of directional influence from the west to the central region was observed, while that in September was found in the southeastern region. However, for most of the northern region, the local accuracy ranges were 0.352–0.444 in July, and 0.239–0.338 in November. 

### 3.6. Comparison between GPR and GWPR

The goodness-of-fit measures for both the GPR and GWPR models are presented in Table 4, revealing that the GWPR model exhibits a smaller AICc value than the GPR model. Conversely, the GWPR model exhibited a higher percentage of deviance than the GPR model. The global percentage of deviance ranged from 0.231 to 0.526, with the highest value observed in January. The residuals of the GWPR model were not statistically significant at a *p* ≤ 0.05, indicating a perfectly random spatial pattern. These findings suggest that the GWPR is a suitable method for elucidating the relationship between the morbidity rate of melioidosis and environmental indicators.

## 4. Discussion

Melioidosis remains an endemic disease in the study area, with cases reported throughout the 10 y study period. Hantrakun et al. [42] found in their study on the clinical epidemiology of 7126 melioidosis patients in 60 hospitals in Thailand from 2012 to 2015 that the Ubon Ratchathani province exhibits a high incidence rate, designating it as a high-risk area. Figure 3 further confirms the occurrence of melioidosis outbreaks, demonstrating that the monthly and annual occurrences of the disease vary, thereby affecting the susceptibility of individuals to melioidosis each month. Given that *B. pseudomallei*, the causative agent of melioidosis, is found in both soil and water, individuals in at-risk populations are more susceptible to infection. Therefore, melioidosis is a significant public health concern that necessitates continuous monitoring, surveillance, and prevention efforts to mitigate its incidence in rural areas.

Because reliance solely on case report data for disease monitoring and surveillance may be insufficient, operations must incorporate spatial data to inform decisions and prevention plans. Spatial autocorrelation analysis using global Moran’s *I* revealed a clustering pattern in the monthly distribution of the melioidosis prevalence. Local spatial correlation analysis using hotspot analysis facilitated the identification of areas with high and low patient numbers, distinguishing between low- and high-risk regions. For instance (Figure 4), a study identified 29 tambons (high–high) as hotspots in January and February, wherein closely clustered locations formed a high-risk group. Conversely, tambons initially deemed low–high may transition into high-risk areas because of their proximity to high-risk groups. Notably, the high-risk group was primarily clustered in the northern region, consistent with the findings of Wongbutdee et al. [21], who identified a significantly elevated incidence of melioidosis in northern Ubon Ratchathani during 2016–2020. Clustering of melioidosis cases suggests heightened exposure to *B. pseudomallei* in these areas [43]. Furthermore, *B. pseudomallei* has been isolated from the environment near patient residences in Northeast Thailand [44], indicating a correlation between the presence of melioidosis cases and *B. pseudomallei* in the surrounding environment. Thus, environmental factors likely contribute to the growth or persistence of *B. pseudomallei* in the soil and water, leading to the occurrence of melioidosis.

This study utilised satellite image data, specifically LST, NDVI, NDWI, and rainfall, to identify the environmental indicators influencing melioidosis occurrence. The analysis was conducted at a local scale, leveraging proximity-based data analysis, which enhanced predictive accuracy. This approach, employed through the GWPR model, outperformed the GPR model, as indicated by smaller AICc values and higher deviances (Table 4). However, the two models serve different purposes. While the GPR model elucidates global-level indicators influencing melioidosis development, the GWPR model highlights local relationships. Analysis of the explanatory variables in August and November, as well as in September and October, demonstrated a strong association between rainfall and melioidosis morbidity rate. Although previous studies have identified this relationship in numerous countries [5,45,46,47,48], it is not significant in Thailand [49]. The GPR model revealed significant associations between rainfall and melioidosis morbidity rate in certain months (e.g., January and August), but not in other months (March, April, May, and June) (Table S1). 

The GPR model can inform policies for the prevention and control of melioidosis at a provincial level. However, its effectiveness is limited owing to variations in local environmental conditions such as temperature, humidity, rainfall, and vegetation. Therefore, satellite image data were also employed to facilitate the surface analysis of land cover, leveraging the ability of satellite images captured using the MODIS platform to monitor environmental changes over time. The GEE is a powerful tool for accessing large geospatial datasets, enabling the analysis and visualisation of geospatial image data in time series, which is instrumental in disease outbreak monitoring and our research, even though only a few studies have been conducted on melioidosis.

The GWPR model utilises the distance weighting of neighbouring locations to estimate the values at the points of interest. The adaptive kernel bandwidth yields a better-fitting model than the fixed kernel employed in this study. The kernel size is determined by the number of observations, with the distance adapted to the density of the nearest neighbours, resulting in a non-uniform spatial weighting shape. This assertion is supported by several previous studies [50,51,52]. The results demonstrate the percentage deviance, explaining the potential relationship between environmental indicators and the morbidity rate of melioidosis in each tambon. Consequently, the coefficients of the best-fitting model indicated the presence of non-stationarity, as evidenced by the different spatial patterns in the local coefficients of each independent variable. Notably, several local coefficients of LST, NDWI, and rainfall were negative, whereas the local coefficient of NDVI was positive (Figures S1–S12). Overall, the contribution of LST showed a negative correlation with the melioidosis morbidity rate despite the association of *B. pseudomallei* contamination in soil and water with temperature. *B. pseudomallei* exhibited its highest growth rate at 37 °C, with modest reductions observed at 30 °C, 40 °C, and 42 °C, and a more pronounced delay at 25 °C [53]. Global warming, characterised by the gradual and persistent increase in the Earth’s atmospheric temperature due to the greenhouse effect, which in turn impacts extreme weather events such as floods and droughts. The sustained rise in temperature may contribute to the adaptation or tolerance of *B. pseudomallei* to stressful environments. Moreover, studies have shown that higher maximum temperatures are associated with an increased risk of melioidosis [46]. The shifting climate has a significant impact on the environment, directly influencing human health and contributing to the rising incidence of melioidosis in regions such as Brazil, as well as parts of Asia, including Thailand and India [54]. Furthermore, factors such as rainfall, humidity, and maximum wind speed have been found to be significantly associated with melioidosis in countries like Singapore, Laos, and Cambodia [5,6].

The NDVI product of the MODIS vegetation indices, produced at 16 d intervals, facilitates consistent spatiotemporal comparisons of vegetation canopy greenness, which is a composite property of the leaf area, chlorophyll content, and canopy structure. During the summer period (mid-February to mid-May), few areas had vegetation cover, primarily dominated by dense paddy fields in the irrigation zones. However, previous studies have identified *B. pseudomallei* activity in soil paddy fields during the dry season [24] and in uncultivated lands [23]. In the rainy season, vegetation cover is increased, comprising paddy fields, grasslands, and forests, which *B. pseudomallei* has been detected in [55,56,57]. Notably, studies have shown no significant differences in *B. pseudomallei* activity between paddy fields and other land use types [57].

Heavy rainfall influences soil moisture and flooding, creating favourable conditions for the presence of *B. pseudomallei* in watershed areas [2,58,59]. NDWI and rainfall exhibited varying coefficients each month, aiding in understanding the spatial distribution of the melioidosis morbidity rate at the local level. The use of satellite imagery enables rapid data acquisition and coverage of large areas, particularly in regions with differing rainfall patterns across tambons. Evaluation of rainfall from CHIRPS involves comparison with gauge observations before application, given its approximately 5 km resolution and large scale [35]. Consequently, the GWPR model assisted in weighting the parameter values for neighbouring observations to generate location-specific estimates. Our study revealed a negative coefficient for rainfall, with nearly half of the associated melioidosis cases displaying high deviance percentages. Nonetheless, an association between melioidosis and rainfall has been reported in various countries, such as Australia [45,46], Taiwan [47], Malaysia [48], and Singapore [5]. Additionally, Shaharudin et al. [60] reported a 1% detection rate of *B. pseudomallei* in soil, indicating a potential risk of melioidosis among flood victims in Kelantan, Malaysia.

**Figure 6:**
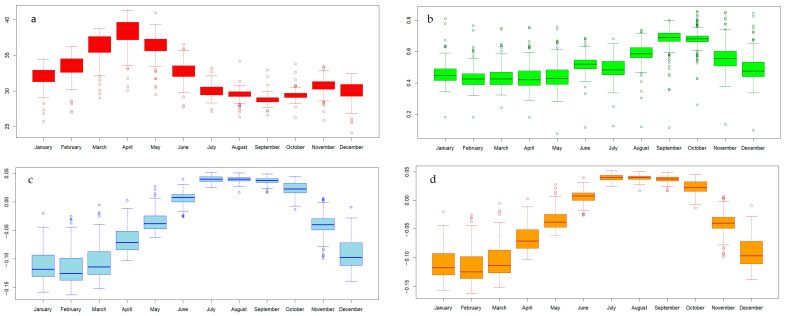
Boxplots of the median and outliers of the explanatory variables in each month for (**a**) LST, (**b**) NDVI, (**c**) NDWI, and (**d**) rainfall.

In the present study, we utilised environmental indicators derived from remotely sensed data to investigate their spatial relationships with the morbidity rate of melioidosis, which differs in spatial heterogeneity in each local area. We employed boxplots to highlight measures of the central tendency of the explanatory variables (Figure 6), identifying outliers in each month and suggesting potential data non-stationarity. This observation indicated an inconsistency in the trend of the monthly melioidosis morbidity rate over the decade, with cases occurring every month. 

However, this study has several limitations. First, the resolution of the satellite imagery of environmental indicators may have affected the scale or resolution of the study area. Therefore, future considerations should incorporate higher-resolution or optimal-scale input data layers, such as Landsat, SPOT, and Sentinel. Second, our retrospective spatiotemporal design may have led to an underestimation of melioidosis cases within the province. Strengthening our study will involve predicting at-risk areas and forecasting melioidosis cases. Finally, while the GWPR model offers moderate reliability, it remains incomplete, similar to other spatial models. Addressing these challenges would involve validating the model performance through training data generation and testing data for validation and accuracy assessment for further research or implementation in public health. Additionally, we suggest further research that includes explanatory factors such as demographic and socioeconomic data, soil texture analysis, and health survey data. Furthermore, investigating the relationship between environmental indicators and bacterial presence in soil and water as well as analysing spatiotemporal data using time-series models will provide valuable insights for future studies.

## 5. Conclusions

This study conducted spatiotemporal analyses of the melioidosis morbidity rate in an endemic area of Ubon Ratchathani over a 10 y period and categorised them into monthly occurrences. The distribution pattern of melioidosis morbidity rate indicated clustering, with hotspots predominantly observed in the northern region. The GPR model proved suitable for studying relationships at the global level and aiding in monitoring and prevention efforts at the provincial level. Meanwhile, the GWPR model was employed to estimate local geographical relationships by utilising nearby location weights to estimate the points of interest, yielding results superior to those of the GPR model. Our findings elucidate the relationship between environmental indicators and the morbidity rate of local melioidosis as indicated by the local percentage of deviance, which assesses the explanatory power of the model in terms of melioidosis occurrence. Although the coefficients for each factor varied from negative to positive, they effectively explained the relative contributions of the LST, NDVI, NDWI, and rainfall to the morbidity rate of melioidosis in each local area. Furthermore, the GWPR model revealed spatial heterogeneity attributable to differences in land surface characteristics and geography across various areas. Consequently, our findings offer valuable insights to guide the delineation of area boundaries when planning melioidosis monitoring and surveillance efforts. Moreover, the methodology employed in this study can be applied to other locations within Thailand and neighbouring countries, given their shared tropical climate characteristics. Furthermore, the utilisation of remotely sensed data, accessible through platforms such as GEE, offers a cost-effective and widely available resource. However, it is imperative to ensure the continuous updating and calibration of information to maintain accuracy, particularly in diverse local contexts.

## Figures and Tables

**Table 1:** Data satellite obtained from Google Earth engine.

ID	Data	Satellite	Data Full Name	Resolution
1	LST	MODIS	MOD11A1.006 Terra Land Surface Temperature and Emissivity Daily Global 1 km	1 km
2	NDVI	MODIS	MOD13Q1.006 Terra Vegetation Indices 16-Day	250 m
3	NDWI	MODIS	MODIS Terra Daily NDWI	463.313 m
4	Rainfall	CHIRPS PENTAD	Climate Hazards Group InfraRed Precipitation with Station Data	5566 m

**Table 2:** Global Moran’s *I* of melioidosis morbidity rate per month.

Monthly	Moran’s *I*	Mean	S.D.	z-Value	Pseudo *p*-Value
January	0.462	–0.004	0.039	11.673	0.002
February	0.317	–0.007	0.040	8.021	0.002
March	0.162	–0.004	0.039	4.178	0.004
April	0.187	–0.007	0.042	4.639	0.002
May	0.068	–0.008	0.038	1.990	0.042
June	0.076	–0.005	0.042	1.913	0.034
July	0.246	–0.006	0.038	6.570	0.002
August	0.351	–0.0003	0.040	8.780	0.002
September	0.253	–0.004	0.040	6.390	0.002
October	0.244	–0.003	0.040	6.083	0.002
November	0.187	–0.006	0.038	4.994	0.002
December	0.257	–0.001	0.039	6.610	0.002

S.D.: Standard Division. pseudo *p* < 0.05.

**Table 3:** Median of generalised geographically weighted Poisson regression (GWPR) coefficient estimates per month.

Monthly	Intercept	LST	NDVI	NDWI	Rainfall
January	–4.546	0.135	1.82	–2.329	0.077
February	–2.289	0.046	–0.055	1.34	0.16
March	2.804	–0.082	–0.193	–6.077	–0.025
April	1.762	–0.054	–3.555	–6.519	0.005
May	–0.546	0.004	–1.581	–10.219	–0.003
June	–2.625	0.073	3.209	4.1483	–0.001
July	5.14	–0.11	–3.195	31.425	0.0008
August	–2.808	0.045	0.72	34.014	0.003
September	2.068	0.033	0.705	–8.659	–0.003
October	–2.948	0.056	6.686	1.9126	–0.006
November	1.991	–0.018	–0.823	4.952	–0.023
December	6.755	–0.208	0.088	–10.567	0.130

**Table 4:** Measures of goodness-of-fit and Moran’s *I* statistics for residuals of GWPR per month.

Monthly	GPR		GWPR		Moran’s *I*	z-Score	*p*-Value
	AICc	Deviance	AICc	Deviance			
January	1197.00	0.144	432.808	0.526	0.008	0.318	0.374
February	924.00	0.157	403.175	0.395	–0.056	–1.275	0.898
March	859.00	0.053	430.776	0.231	–0.019	–0.373	0.645
April	828.00	0.025	431.394	0.280	–0.035	–0.753	0.774
May	850.00	0.037	436.957	0.145	–0.025	–0.520	0.698
June	952.00	0.018	441.450	0.299	–0.104	–2.435	0.992
July	974.00	0.204	406.892	0.325	–0.072	–1.656	0.951
August	1087.00	0.121	421.122	0.416	–0.048	–1.071	0.858
September	1075.00	0.087	439.833	0.442	–0.092	–2.154	0.984
October	899.00	0.132	408.941	0.378	–0.047	–1.059	0.855
November	837.00	0.022	418.079	0.275	–0.035	–0.747	0.772
December	751.00	0.156	402.712	0.422	0.014	0.475	0.317

AICc: Akaike information criterion. Deviance: percentage of deviance.

## Data Availability

Data of this study are contained within the article or Supplementary Materials.

## Supplementary Materials

The following supporting information can be downloaded at: https://www.mdpi.com/article/10.3390/ijerph21050614/s1, Figure S1: The spatial variation in the coefficients of GWPR model in January, Figure S2: The spatial variation in the coefficients of GWPR model in February, Figure S3: The spatial variation in the coefficients of GWPR model in March, Figure S4: The spatial variation in the coefficients of GWPR model in April, Figure S5: The spatial variation in the coefficients of GWPR model in May, Figure S6: The spatial variation in the coefficients of GWPR model in June, Figure S7: The spatial variation in the coefficients of GWPR model in July, Figure S8: The spatial variation in the coefficients of GWPR model in August, Figure S9: The spatial variation in the coefficients of GWPR model in September, Figure S10: The spatial variation in the coefficients of GWPR model in October, Figure S11: The spatial variation in the coefficients of GWPR model in November, Figure S12: The spatial variation in the coefficients of GWPR model in December; Table S1: Results of parameter estimates in the GPR and GWPR per Month.

## References

[1] Currie B.J., Dance D.A., Cheng A.C. (2008). The global distribution of Burkholderia pseudomallei and melioidosis: An update. Trans. R. Soc. Trop. Med. Hyg..

[2] Corkeron M.L., Norton R., Nelson P.N. (2010). Spatial analysis of melioidosis distribution in a suburban area. Epidemiol. Infect..

[3] Limmathurotsakul D., Golding N., Dance D.A., Messina J.P., Pigott D.M., Moyes C.L., Rolim D.B., Bertherat E., Day N.P., Peacock S.J. (2016). Predicted global distribution of Burkholderia pseudomallei and burden of melioidosis. Nat. Microbiol..

[4] Wiersinga W.J., Virk H.S., Torres A.G., Currie B.J., Peacock S.J., Dance D.A.B., Limmathurotsakul D. (2018). Melioidosis. Nat. Rev. Dis. Primers.

[5] Liu X., Pang L., Sim S.H., Goh K.T., Ravikumar S., Win M.S., Tan G., Cook A.R., Fisher D., Chai L.Y. (2015). Association of melioidosis incidence with rainfall and humidity, Singapore, 2003–2012. Emerg. Infect. Dis..

[6] Bulterys P.L., Bulterys M.A., Phommasone K., Luangraj M., Mayxay M., Kloprogge S., Miliya T., Vongsouvath M., Newton P.N., Phetsouvanh R. (2018). Climatic drivers of melioidosis in Laos and Cambodia: A 16-year case series analysis. Lancet Planet Health.

[7] Kaewpan A., Duangurai T., Rungruengkitkun A., Muangkaew W., Kanjanapruthipong T., Jitprasutwit N., Ampawong S., Sukphopetch P., Chantratita N., Pumirat P. (2022). Burkholderia pseudomallei pathogenesis in human skin fibroblasts: A Bsa type III secretion system is involved in the invasion, multinucleated giant cell formation, and cellular damage. PLoS ONE.

[8] Limmathurotsakul D., Dance D.A., Wuthiekanun V., Kaestli M., Mayo M., Warner J., Wagner D.M., Tuanyok A., Wertheim H., Yoke Cheng T. (2013). Systematic review and consensus guidelines for environmental sampling of Burkholderia pseudomallei. PLoS Negl. Trop. Dis..

[9] Chen Y.S., Chen S.C., Kao C.M., Chen Y.L. (2003). Effects of soil pH, temperature and water content on the growth of Burkholderia pseudomallei. Folia Microbiol..

[10] Palasatien S., Lertsirivorakul R., Royros P., Wongratanacheewin S., Sermswan R.W. (2008). Soil physicochemical properties related to the presence of Burkholderia pseudomallei. Trans. R. Soc. Trop. Med. Hyg..

[11] Tong S., Yang S., Lu Z., He W. (1996). Laboratory investigation of ecological factors influencing the environmental presence of Burkholderia pseudomallei. Microbiol. Immunol..

[12] McMichael A.J., Lindgren E. (2011). Climate change: Present and future risks to health, and necessary responses. J. Intern. Med..

[13] Guo Y., Gasparrini A., Armstrong B.G., Tawatsupa B., Tobias A., Lavigne E., Coelho M., Pan X., Kim H., Hashizume M. (2017). Heat Wave and Mortality: A Multicountry, Multicommunity Study. Environ. Health Perspect..

[14] Chai L.Y.A., Fisher D. (2018). Earth, wind, rain, and melioidosis. Lancet Planet. Health.

[15] Kamal A., Al-Montakim M.N., Hasan M.A., Mitu M.M.P., Gazi M.Y., Uddin M.M., Mia M.B. (2023). Relationship between Urban Environmental Components and Dengue Prevalence in Dhaka City-An Approach of Spatial Analysis of Satellite Remote Sensing, Hydro-Climatic, and Census Dengue Data. Int. J. Environ. Res. Public Health.

[16] Sewe M.O., Ahlm C., Rocklöv J. (2016). Remotely Sensed Environmental Conditions and Malaria Mortality in Three Malaria Endemic Regions in Western Kenya. PLoS ONE.

[17] Dhewantara P.W., Hu W., Zhang W., Yin W.W., Ding F., Mamun A.A., Soares Magalhães R.J. (2019). Climate variability, satellite-derived physical environmental data and human leptospirosis: A retrospective ecological study in China. Environ. Res..

[18] Goodrick I., Stewart J., Todd G. (2018). Soil characteristics influencing the spatial distribution of melioidosis in Far North Queensland, Australia. Epidemiol. Infect..

[19] Kanjaras P., Bumrerraj S., Seng R., Noradee S., Nithikathkul C. (2023). Geospatial Analysis and Modeling of Melioidosis Prevention and Control in Si Sa Ket Province, Thailand. J. Geoinform..

[20] Wongbutdee J., Saengnill W., Jittimanee J., Panomket P., Saenwang P. (2021). The Association between the Mapping Distribution of Melioidosis Incidences and Meteorological Factors in an Endemic Area: Ubon Ratchathani, Thailand (2009–2018). Chiang Mai Univ. (CMU) J. Nat. Sci..

[21] Wongbutdee J., Jittimanee J., Saengnill W. (2023). Spatiotemporal distribution and geostatistically interpolated mapping of the melioidosis risk in an endemic zone in Thailand. Geospat. Health.

[22] Wuthiekanun V., Mayxay M., Chierakul W., Phetsouvanh R., Cheng A.C., White N.J., Day N.P., Peacock S.J. (2005). Detection of Burkholderia pseudomallei in soil within the Lao People’s Democratic Republic. J. Clin. Microbiol..

[23] Limmathurotsakul D., Wuthiekanun V., Chantratita N., Wongsuvan G., Amornchai P., Day N.P.J., Peacock S.J. (2010). Burkholderia pseudomallei Is Spatially Distributed in Soil in Northeast Thailand. PLoS Neglected Trop. Dis..

[24] Saengnill W., Charoenjit K., Hrimpeng K., Jittimanee J. (2020). Mapping the probability of detecting Burkholderia pseudomallei in rural rice paddy soil based on indicator kriging and spatial soil factor analysis. Trans. R. Soc. Trop. Med. Hyg..

[25] Wimberly M.C., Nekorchuk D.M., Kankanala R.R. (2022). Cloud-based applications for accessing satellite Earth observations to support malaria early warning. Sci. Data.

[26] Li Z., Gurgel H., Xu L., Yang L., Dong J. (2022). Improving Dengue Forecasts by Using Geospatial Big Data Analysis in Google Earth Engine and the Historical Dengue Information-Aided Long Short Term Memory Modeling. Biology.

[27] Ghosh S., Kumar D., Kumari R. (2022). Google earth engine based computational system for the earth and environment monitoring applications during the COVID-19 pandemic using thresholding technique on SAR datasets. Phys. Chem. Earth.

[28] Nafiz Rahaman S., Shehzad T., Sultana M. (2022). Effect of Seasonal Land Surface Temperature Variation on COVID-19 Infection Rate: A Google Earth Engine-Based Remote Sensing Approach. Environ. Health Insights.

[29] Bureau of Epidemiology, Thailand National Disease Surveillance (Report 506): Melioidosis. http://doe.moph.go.th/surdata/y62/rate_Melioidosis_62.rtf.

[30] Bureau of Epidemiology, Thailand National Disease Surveillance (Report 506): Melioidosis. http://doe.moph.go.th/surdata/y63/mcd_Melioidosis_63.rtf.

[31] Tennekes M. (2018). tmap: Thematic Maps in R. J. Stat. Softw..

[32] Didan K., Barreto-Muñoz A. MODIS Collection 6.1 (C61) VegetationIndex Product UserGuide. https://lpdaac.usgs.gov/documents/621/MOD13_User_Guide_V61.pdf.

[33] Gao B.-c. (1996). NDWI—A normalized difference water index for remote sensing of vegetation liquid water from space. Remote Sens. Environ..

[34] Ceccato P., Flasse S., Tarantola S., Jacquemoud S., Grégoire J.-M. (2001). Detecting vegetation leaf water content using reflectance in the optical domain. Remote Sens. Environ..

[35] Funk C., Peterson P., Landsfeld M., Pedreros D., Verdin J., Shukla S., Husak G., Rowland J., Harrison L., Hoell A. (2015). The climate hazards infrared precipitation with stations--a new environmental record for monitoring extremes. Sci. Data.

[36] Chaithong T. (2022). Flash Flood Susceptibility Assessment Based on Morphometric Aspects and Hydrological Approaches in the Pai River Basin, Mae Hong Son, Thailand. Water.

[37] Puttanapong N., Martinez A., Bulan J.A., Addawe M., Durante R.L., Martillan M. (2022). Predicting Poverty Using Geospatial Data in Thailand. ISPRS Int. J. Geo-Inf..

[38] Rojpratak S., Supharatid S. (2023). Regional-scale flood impacts on a small mountainous catchment in Thailand under a changing climate. J. Water Clim. Chang..

[39] Nakaya T., Fotheringham A.S., Brunsdon C., Charlton M. (2005). Geographically weighted Poisson regression for disease association mapping. Stat. Med..

[40] Fotheringham A.S., Brunsdon C., Charlton M. (2003). Geographically Weighted Regression: The Analysis of Spatially Varying Relationships.

[41] Hadayeghi A., Shalaby A., Persaud B. (2010). Development of Planning-Level Transportation Safety Models using Full Bayesian Semiparametric Additive Techniques. J. Transp. Saf. Secur..

[42] Hantrakun V., Kongyu S., Klaytong P., Rongsumlee S., Day N.P.J., Peacock S.J., Hinjoy S., Limmathurotsakul D. (2019). Clinical Epidemiology of 7126 Melioidosis Patients in Thailand and the Implications for a National Notifiable Diseases Surveillance System. Open Forum Infect. Dis..

[43] Dai D., Chen Y.S., Chen P.S., Chen Y.L. (2012). Case cluster shifting and contaminant source as determinants of melioidosis in Taiwan. Trop. Med. Int. Health.

[44] Seng R., Saiprom N., Phunpang R., Baltazar C.J., Boontawee S., Thodthasri T., Silakun W., Chantratita N. (2019). Prevalence and genetic diversity of Burkholderia pseudomallei isolates in the environment near a patient’s residence in Northeast Thailand. PLoS Neglected Trop. Dis..

[45] Currie B.J., Jacups S.P. (2003). Intensity of rainfall and severity of melioidosis, Australia. Emerg. Infect. Dis..

[46] Kaestli M., Grist E.P.M., Ward L., Hill A., Mayo M., Currie B.J. (2016). The association of melioidosis with climatic factors in Darwin, Australia: A 23-year time-series analysis. J. Infect..

[47] Mu J.J., Cheng P.Y., Chen Y.S., Chen P.S., Chen Y.L. (2014). The occurrence of melioidosis is related to different climatic conditions in distinct topographical areas of Taiwan. Epidemiol. Infect..

[48] Jiee S., Lim K., Choon Vui D., Marius D., Illyana N., Jantim A. (2023). Extreme Weather and Melioidosis: An endemic tropical disease in Penampang district of Sabah, Malaysia. J. Health Res..

[49] Limmathurotsakul D., Wongratanacheewin S., Teerawattanasook N., Wongsuvan G., Chaisuksant S., Chetchotisakd P., Chaowagul W., Day N.P., Peacock S.J. (2010). Increasing incidence of human melioidosis in Northeast Thailand. Am. J. Trop. Med. Hyg..

[50] John Braun W., Rousson V. (2000). An autocorrelation criterion for bandwidth selection in nonparametric regression. J. Stat. Comput. Simul..

[51] Tavares J.P., Costa A.C. (2021). Spatial Modeling and Analysis of the Determinants of Property Crime in Portugal. ISPRS Int. J. Geo-Inf..

[52] Zeleke A.J., Miglio R., Palumbo P., Tubertini P., Chiari L. (2022). Spatiotemporal heterogeneity of SARS-CoV-2 diffusion at the city level using geographically weighted Poisson regression model: The case of Bologna, Italy. Geospat. Health.

[53] Paksanont S., Sintiprungrat K., Yimthin T., Pumirat P., Peacock S.J., Chantratita N. (2018). Effect of temperature on Burkholderia pseudomallei growth, proteomic changes, motility and resistance to stress environments. Sci. Rep..

[54] Birnie E., Biemond J.J., Wiersinga W.J. (2022). Drivers of melioidosis endemicity: Epidemiological transition, zoonosis, and climate change. Curr. Opin. Infect. Dis..

[55] Kaestli M., Mayo M., Harrington G., Ward L., Watt F., Hill J.V., Cheng A.C., Currie B.J. (2009). Landscape changes influence the occurrence of the melioidosis bacterium Burkholderia pseudomallei in soil in northern Australia. PLoS Neglected Trop. Dis..

[56] Ribolzi O., Rochelle-Newall E., Dittrich S., Auda Y., Newton P.N., Rattanavong S., Knappik M., Soulileuth B., Sengtaheuanghoung O., Dance D.A. (2016). Land use and soil type determine the presence of the pathogen Burkholderia pseudomallei in tropical rivers. Environ. Sci. Pollut. Res. Int..

[57] Shaw T., Assig K., Tellapragada C., Wagner G.E., Choudhary M., Göhler A., Eshwara V.K., Steinmetz I., Mukhopadhyay C. (2022). Environmental Factors Associated with Soil Prevalence of the Melioidosis Pathogen Burkholderia pseudomallei: A Longitudinal Seasonal Study from South West India. Front. Microbiol..

[58] Kaestli M., Mayo M., Harrington G., Watt F., Hill J., Gal D., Currie B.J. (2007). Sensitive and specific molecular detection of Burkholderia pseudomallei, the causative agent of melioidosis, in the soil of tropical northern Australia. Appl. Environ. Microbiol..

[59] Chuah C.J., Tan E.K.H., Sermswan R.W., Ziegler A.D. (2017). Hydrological connectivity and Burkholderia pseudomallei prevalence in wetland environments: Investigating rice-farming community’s risk of exposure to melioidosis in North-East Thailand. Environ. Monit. Assess..

[60] Shaharudin R., Ahmad N., Kamaluddin M.A., Veloo Y. (2016). Detection of burkholderia pseudomallei from post-flood soil samples in kelantan, malaysia. Southeast Asian J. Trop. Med. Public Health.

