# Supplementary material for: Exploring the Relationship between Melioidosis Morbidity Rate and Local Environmental Indicators Using Remotely Sensed Data
Source: Int J Environ Res Public Health. 2024 May 13;21(5):614. doi: 10.3390/ijerph21050614 (PMC11121278; doi:10.3390/ijerph21050614)
Supplement: Supplementary file 1 [file ijerph-21-00614-s001.zip › ijerph-2974735-supplementary.pdf]

Supplementary Information

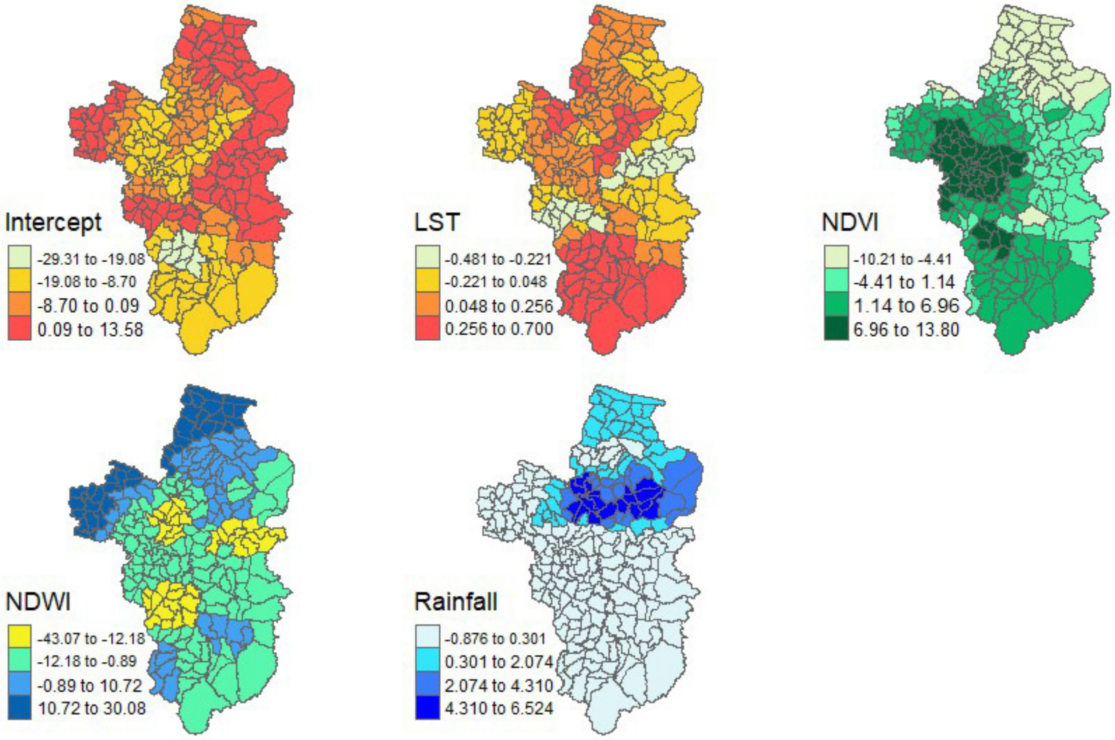

Figure S1. The spatial variation of the coefficients of GWPR model in January.

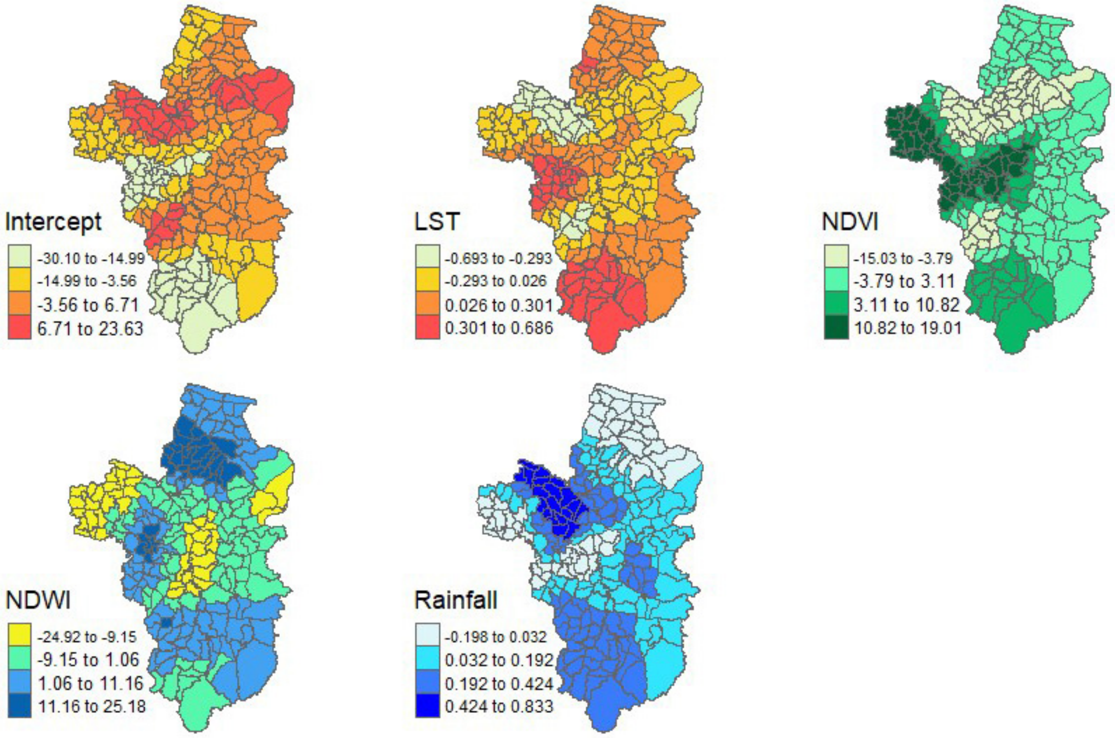

Figure S2. The spatial variation of the coefficients of GWPR model in February**.**

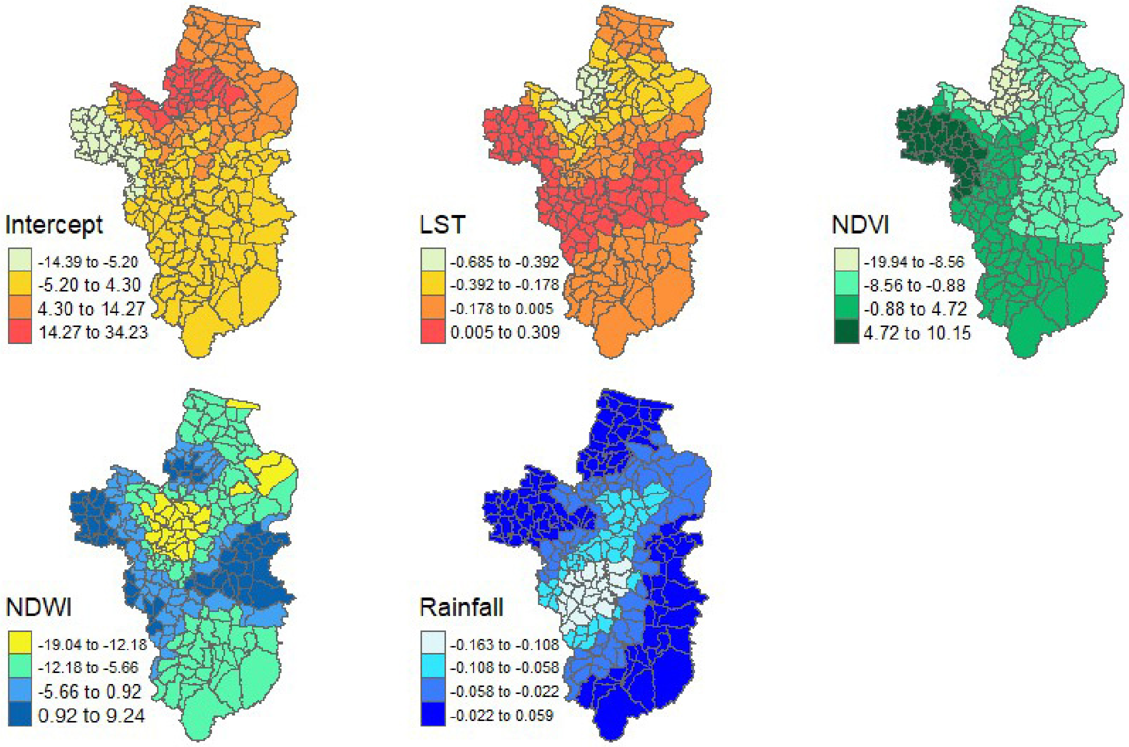

Figure S3. The spatial variation of the coefficients of GWPR model in March.

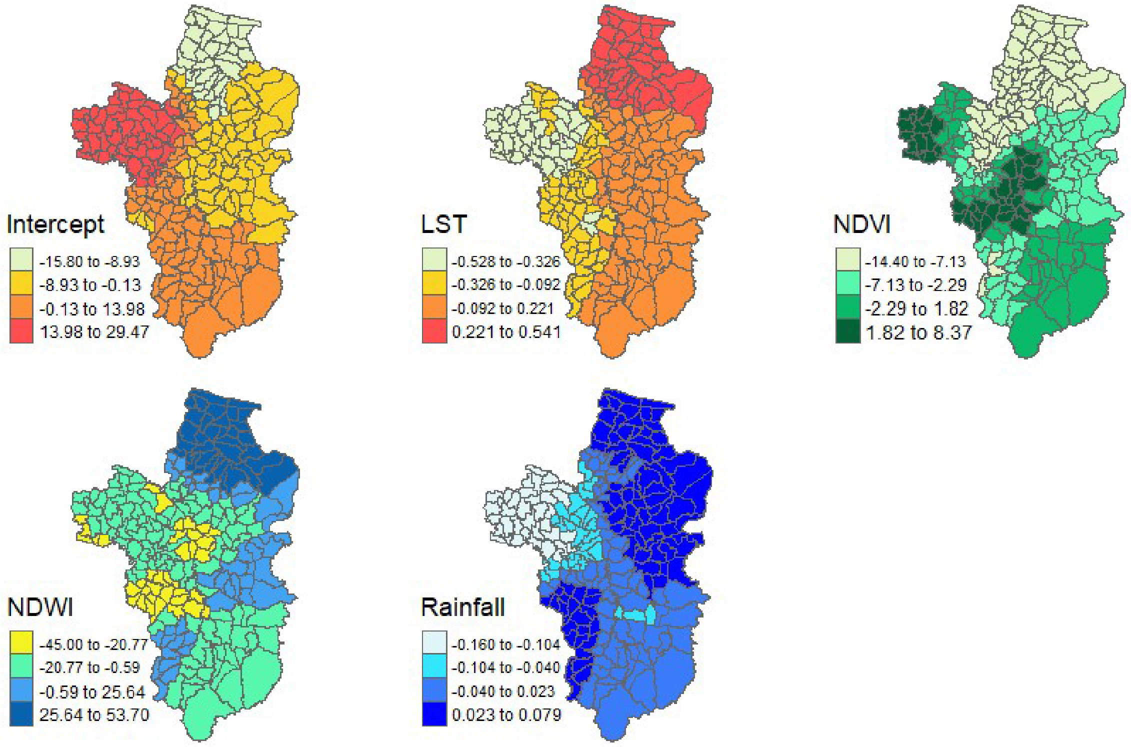

Figure S4. The spatial variation of the coefficients of GWPR model in April.

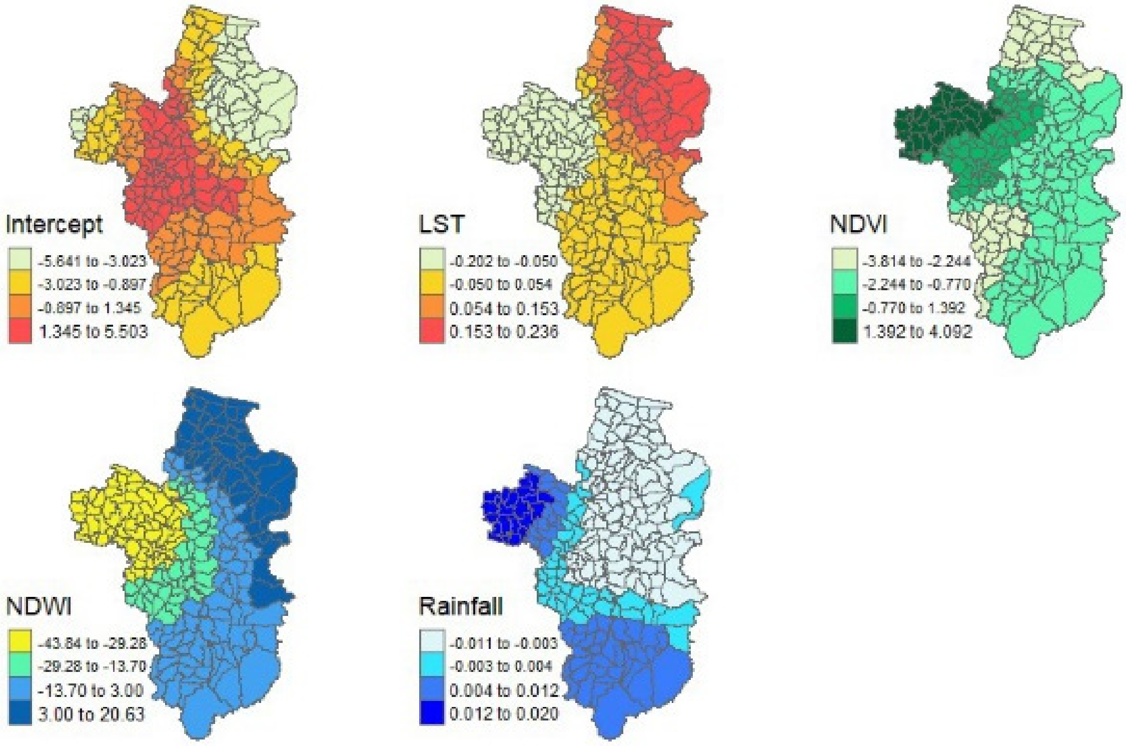

Figure S5. The spatial variation of the coefficients of GWPR model in May.

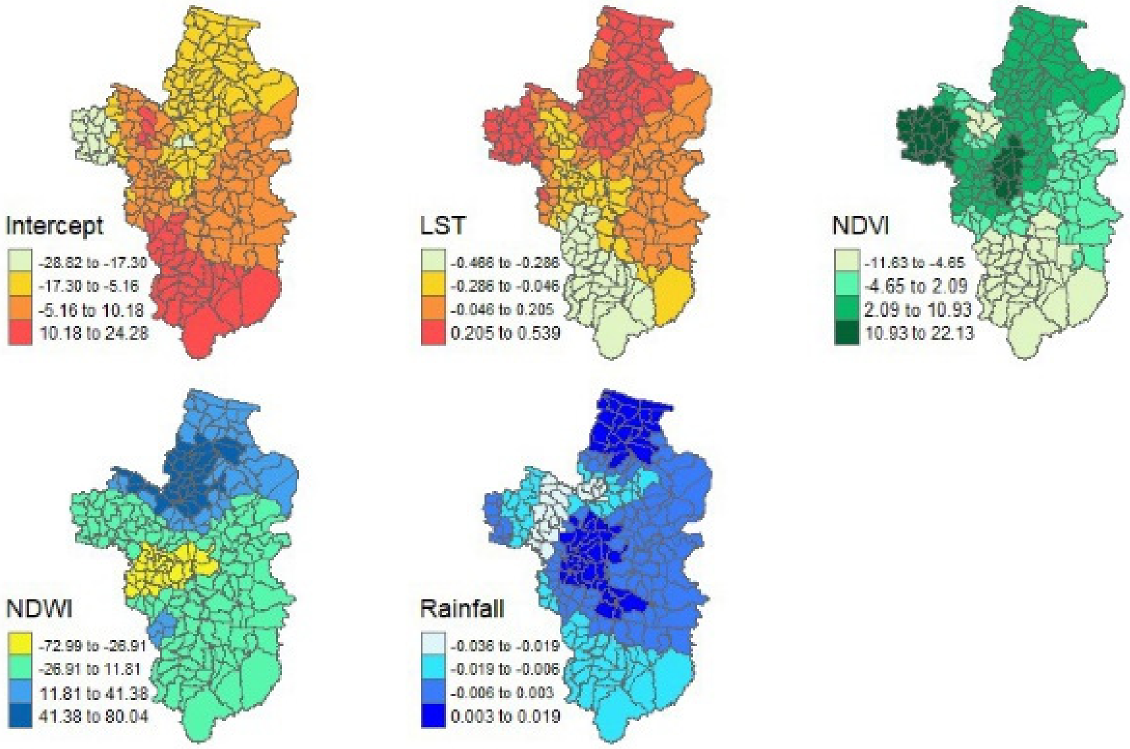

Figure S6. The spatial variation of the coefficients of GWPR model in June.

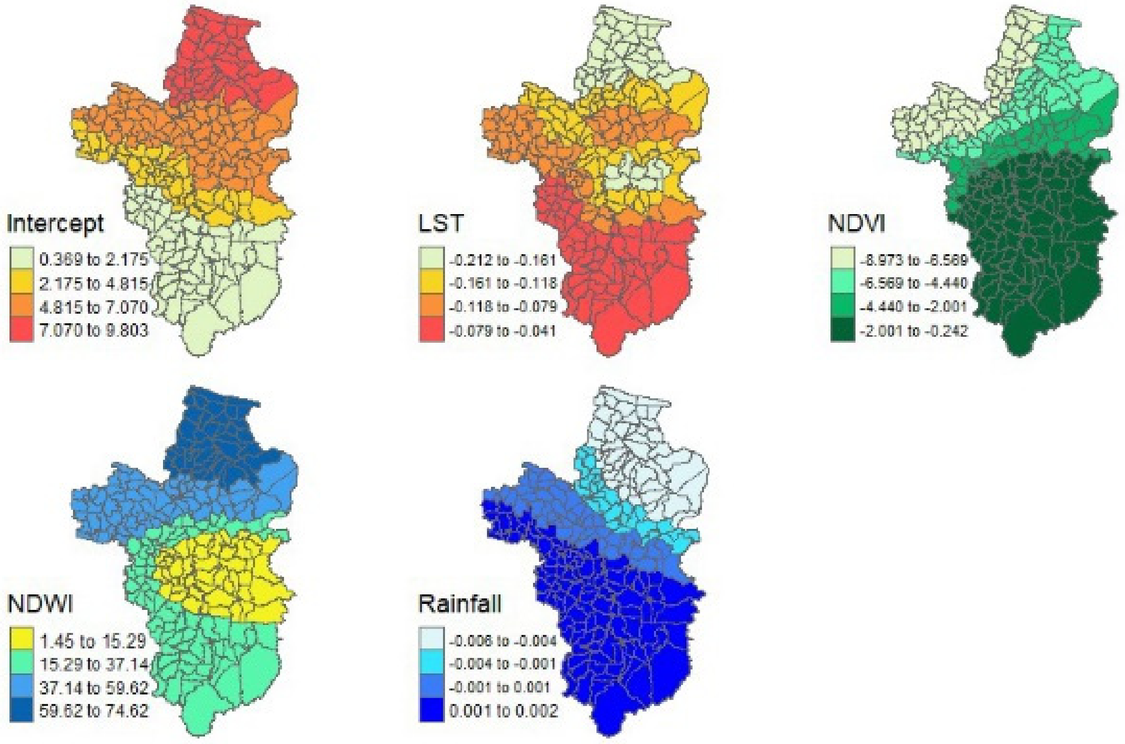

Figure S7. The spatial variation of the coefficients of GWPR model in July.

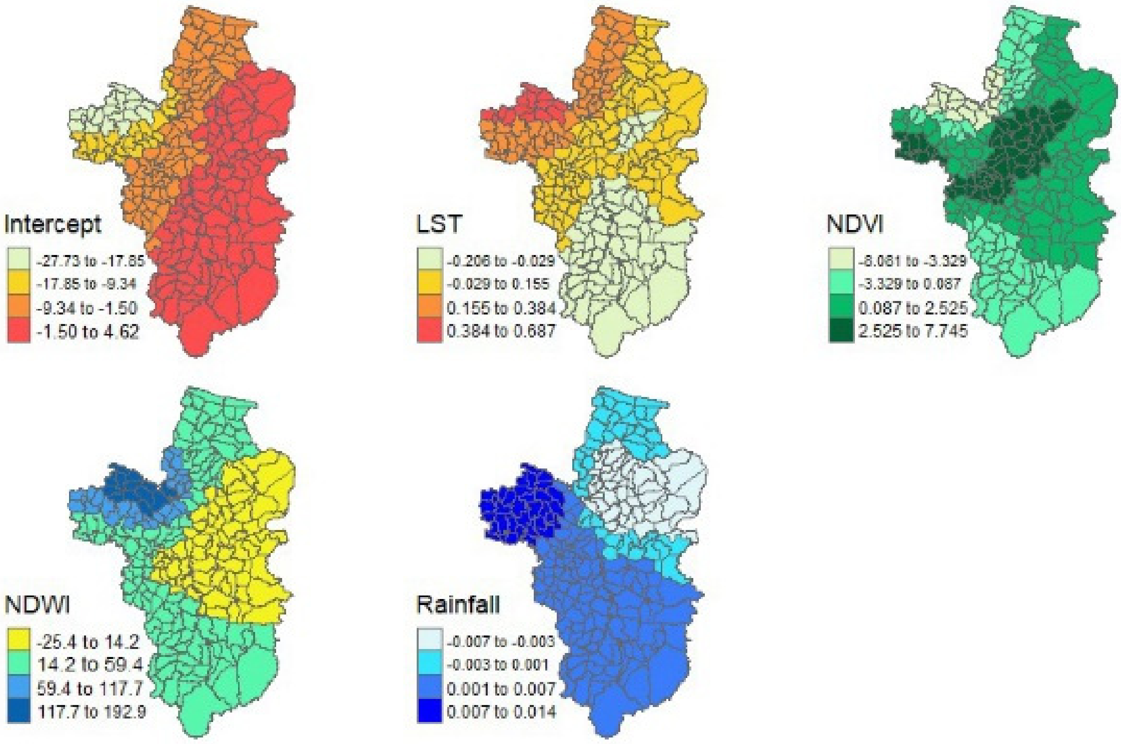

Figure S8. The spatial variation of the coefficients of GWPR model in August.

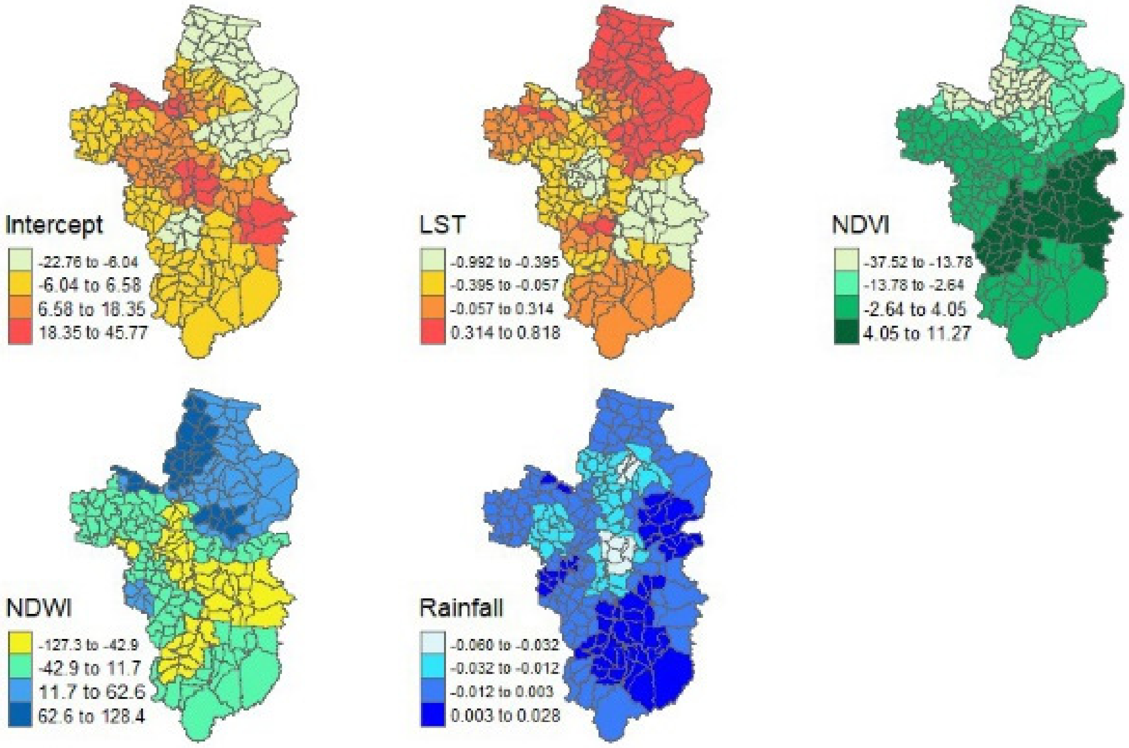

Figure S9. The spatial variation of the coefficients of GWPR model in September.

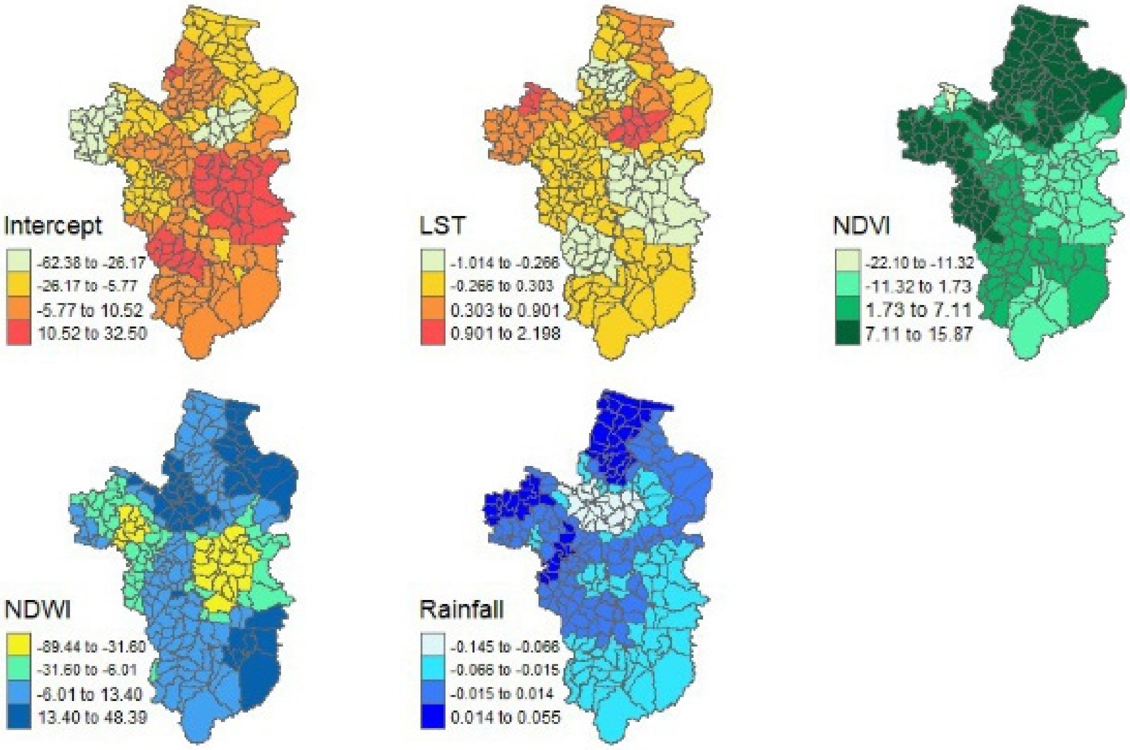

Figure S10. The spatial variation of the coefficients of GWPR model in October.

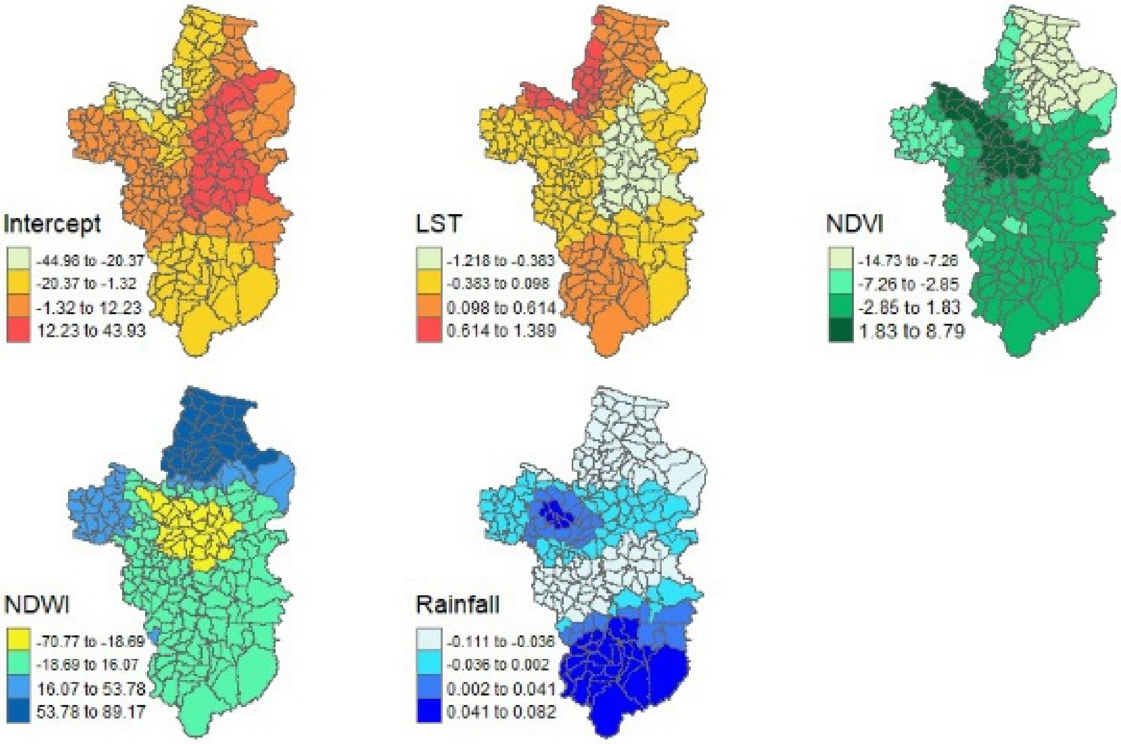

Figure S11. The spatial variation of the coefficients of GWPR model in November.

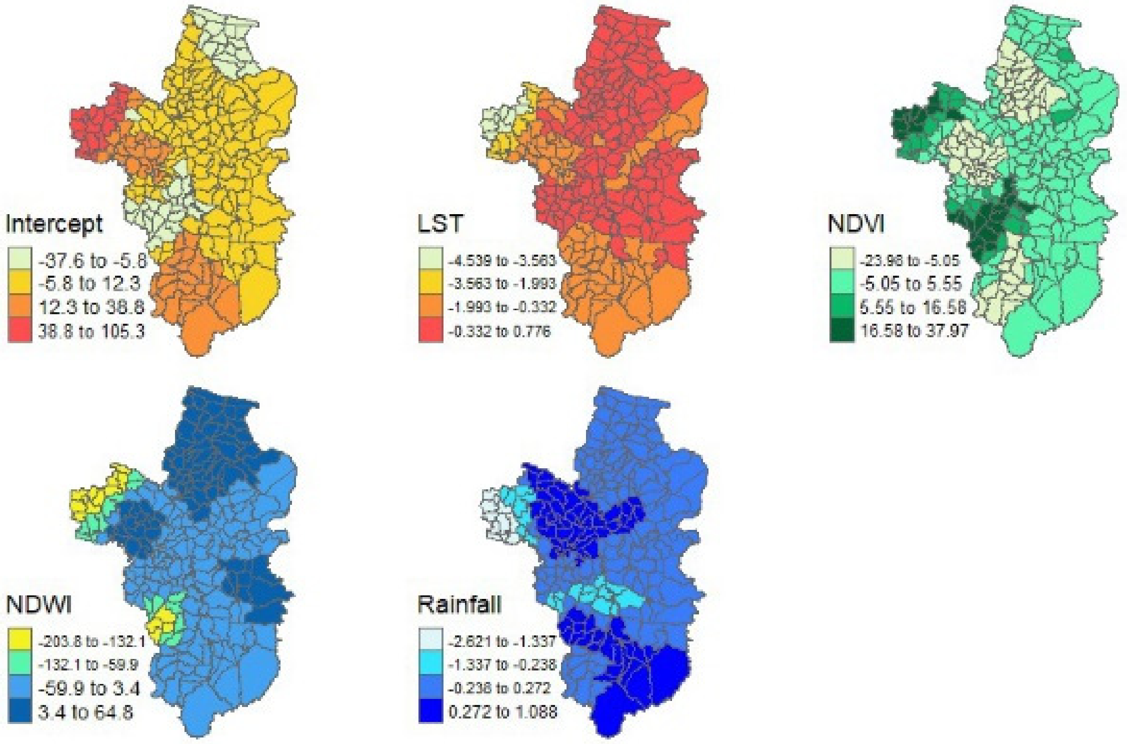

Figure S12. The spatial variation of the coefficients of GWPR model in December.

Table S1. Results of parameter estimates in the GPR and GWPR per Month.

Variable	GPR					GWPR		
	Estimate	Std. Error	z value	P-value	VIF	Minimum	Median	Maximum
**January**								
Intercept	4.035	1.388	2.906	0.003*	-	-29.306	-4.546	13.577
LST_1	-0.091	0.047	-1.903	0.056	3.315	-0.480	0.135	0.700
NDVI_1	-1.852	0.692	-2.676	0.007*	1.746	-10.206	1.820	13.798
NDWI_1	-11.095	2.630	-4.217	<0.0001*	3.054	-43.066	-2.329	30.076
RAIN_1	-0.178	0.031	-5.746	<0.0001*	1.143	-0.876	0.077	6.524
**February**								
Intercept	-0.300	1.999	-0.150	0.880	-	-30.095	-2.289	23.633
LST_2	-0.018	0.061	-0.295	0.767	3.496	-0.692	0.046	0.686
NDVI_2	-1.322	0.987	-1.339	0.180	1.758	-15.031	-0.055	19.014
NDWI_2	-3.014	3.775	-0.798	0.424	4.292	-24.921	1.340	25.184
RAIN_2	0.212	0.032	6.479	<0.0001*	1.250	-0.197	0.160	0.833
**March**								
Intercept	6.135	1.974	3.107	0.001*	-	-14.387	2.804	34.225
LST_3	-0.157	0.060	-2.603	0.009*	3.804	-0.685	-0.082	0.308
NDVI_3	-1.265	1.122	-1.127	0.259	2.191	-19.936	-0.193	10.151
NDWI_3	-12.481	4.072	-3.064	0.002*	4.340	-19.037	-6.077	9.244
RAIN_3	-0.023	0.014	-1.626	0.103	1.033	-0.162	-0.025	0.058
**April**								
Intercept	-2.185	2.127	-1.027	0.304	-	-15.795	1.762	29.470
LST_4	0.110	0.061	1.784	0.074	3.822	-0.527	-0.054	0.541
NDVI_4	-1.615	1.244	-1.298	0.194	2.342	-14.398	-3.555	8.367
NDWI_4	4.658	6.133	0.759	0.447	5.096	-44.996	-6.519	53.702
RAIN_4	-0.010	0.011	-0.900	0.367	1.113	-0.160	0.005	0.078
**May**								
Intercept	1.929	2.270	0.850	0.395	-	-5.640	-0.546	5.502
LST_5	0.003	0.054	0.069	0.944	2.459	-0.201	0.004	0.236
NDVI_5	-1.831	0.982	-1.863	0.062	2.420	-3.813	-1.581	4.092
NDWI_5	-4.794	5.942	-0.806	0.419	3.757	-43.837	-10.219	20.632
RAIN_5	-0.003	0.003	-1.136	0.255	1.242	-0.011	-0.003	0.020
**June**								
Intercept	1.294	1.620	0.798	0.424	-	-28.823	-2.625	24.282
LST_6	0.019	0.043	0.449	0.653	1.607	-0.466	0.073	0.539
NDVI_6	-1.499	0.745	-2.011	0.044*	1.088	-11.629	3.209	22.125
NDWI_6	12.079	5.352	2.256	0.024*	1.573	-72.993	4.1483	80.041
RAIN_6	-0.001	0.001	-1.403	0.160	1.326	-0.035	-0.001	0.018
**July**								
Intercept	5.593	1.882	2.971	0.002*	-	0.368	5.140	9.802
LST_7	-0.143	0.059	-2.412	0.015*	1.330	-0.211	-0.110	-0.040
NDVI_7	-4.842	0.605	-7.994	<0.0001*	1.187	-8.973	-3.195	-0.241
NDWI_7	64.235	8.055	7.974	<0.0001*	1.134	1.447	31.425	74.617
RAIN_7	-0.001	0.0007	-1.950	0.051	1.322	-0.005	0.0008	0.002
**August**								
Intercept	-8.249	1.928	-4.277	<0.0001*	-	-27.725	-2.808	4.620

LST_8	0.237	0.056	4.207	<0.0001*	1.240	-0.206	0.045	0.687
NDVI_8	-2.874	0.625	-4.595	<0.0001*	1.167	-8.081	0.720	7.744
NDWI_8	72.880	9.152	7.963	<0.0001*	1.238	-25.417	34.014	192.858
RAIN_8	0.002	0.0006	4.110	<0.0001*	1.248	-0.006	0.003	0.014
**September**								
Intercept	1.372	2.643	0.519	0.603	-	-22.759	2.068	45.772
LST_9	0.000	0.079	0.001	0.998	1.258	-0.992	0.033	0.817
NDVI_9	3.673	0.910	4.034	<0.0001*	1.313	-37.523	0.705	11.266
NDWI_9	-1.215	8.055	-0.150	0.880	1.450	-127.278	-8.659	128.350
RAIN_9	-0.007	0.001	-5.269	<0.0001*	1.137	-0.059	-0.003	0.028
**October**								
Intercept	6.709	3.362	1.995	0.046*	-	-62.382	-2.948	32.500
LST_10	-0.178	0.100	-1.774	0.075	1.688	-1.013	0.056	2.198
NDVI_10	1.474	0.968	1.522	0.127	1.331	-22.095	6.686	15.871
NDWI_10	2.153	4.884	0.440	0.659	1.578	-89.435	1.9126	48.391
RAIN_10	-0.019	0.002	-7.456	<0.0001*	1.251	-0.144	-0.006	0.054
**November**								
Intercept	6.705	2.725	2.460	0.013*	-	-44.975	1.991	43.933
LST_11	-0.167	0.084	-1.991	0.046*	2.354	-1.218	-0.018	1.389
NDVI_11	-1.119	0.830	-1.347	0.177	2.036	-14.731	-0.823	8.788
NDWI_11	-1.603	4.932	-0.324	0.745	2.564	-70.773	4.952	89.168
RAIN_11	-0.035	0.012	-2.949	0.003*	1.189	-0.110	-0.023	0.082
**December**								
Intercept	1.181	2.567	0.459	0.645	-	-37.627	6.755	105.256
LST_12	-0.134	0.085	-1.571	0.115	2.803	-4.539	-0.208	0.775
NDVI_12	2.214	1.253	1.767	0.077	2.043	-23.980	0.088	37.968
NDWI_12	-12.617	5.445	-2.317	0.020*	3.885	-203.774	-10.567	64.757
RAIN_12	0.193	0.025	7.728	<0.0001*	1.157	-2.620	0.130	1.087
